# New Jersey State Dental Society

**Published:** 1889-02

**Authors:** 


### NEW JERSEY STATE DENTAL SOCIETY.

JULY, 1888.

ESPECIALLY REPORTED FOR THE INTERNATIONAL.
DISCUSSION ON DR. CURTIS' PAPER ON IMPLANTATION.

*Dr. E. C. Kirk, Phila.*—Mr. President, unfortunately I was not present when the first part of Dr. Curtis' paper was read. The subject of implantation is one that interests me a great deal. The first case that I had an opportunity to operate upon was in November, 1886. That case, together with thirty-eight others up to-day, have been entirely satisfactory, both to myself and to the patients for whom I have operated. There have been two instances in which I have lost implanted teeth. One was reported in the *Dental Cosmos* some time ago, and was where I implanted two teeth for a patient who had been suffering from secondary syphilis, which I was not aware of at the time. A more recent one in the mouth of a gentleman whom I took to be a typically favorable case for the operation; he was free from all constitutional vice whatsoever, and was a man of fine physique and apparently in excellent health. The tooth was selected with the same care as the other, and I am entirely at a loss to account for the failure of the operation, which occurred five or six months afterwards by absorption of the root.

We are all sufficiently familiar with the fact that teeth can become united with the alveolar process in this method of implantation introduced by Dr. Younger. The point of special interest is, how long will they last, and what are the conditions necessary to the permanency of the operation. The investigations made by our histologists, Drs. Miller, Heitzmann, Sudduth and others in this direction, seem to show that these implanted teeth are held in place or attached simply by the process of encapsulation, and I think it is unfortunate that Dr. Younger propounded the theory that the peridental membrane would become revitalized after having been separated from its normal surroundings for a considerable length of time, for that theory seems to have been disproven.

The question of the length of time an extracted tooth can remain out of the mouth and be successfully implanted is an interesting one. I have implanted teeth that had been out of the mouth from a year to eighteen months. Within the past six months I have had a letter from my friend D. H. C. Herring of Concord, North Carolina, for whom I performed this operation, stating that he has since then, in his own practice, implanted a number of teeth, among them an incisor tooth which had been extracted seventeen years previously, union taking place without difficulty. The question of the revitalization of the peridental membrane in that case would seem to be entirely eliminated. It seems unreasonable to suppose that the membrane can retain its vitality for any great length of time. I believe that a few hours settles the question of its vitality. Theoretically it seems possible that a tooth devoid of peridental membrane may become attached after implantation. I do not mean to say it will be attached as readily, or that the conditions are just as favorable; but I see no reason why a tooth denuded of its pericementum should not become attached if the process of attachment is one of encapsulation of the root. My belief is that the old dry membrane presents a condition that is more acceptable to the tissues around an implanted tooth, which are more or less irritated by the presence of that foreign body. I think the whole secret of the successful results depends upon controlling the inflammatory process following the operation, so that it shall not at any time exceed the amount necessary to produce repair of the tissue. If the inflammation becomes excessive, nature makes an effort to get rid of the cause, and pus is formed. To avoid this result it is necessary to use, as Dr. Younger did, thorough antiseptic precaution to keep the inflammatory process within the limits necessary to bring about encapsulation.

With regard to the ultimate success of the operation, the two cases I have mentioned have, I think, considerable bearing. It has been generally believed that we should not attempt an operation in cases of scrofulous diathesis, or where there is any systemic vice, such as syphilis, a strumous condition, tuberculosis, or during the period of gestation; yet I have had to report a case of failure in the mouth of a patient whom I believed to be in typically good health, the implanted tooth being lost by absorption of the root. I found that the root was almost completely absorbed. It is important to know why, in such a healthy and favorable looking case, absorption of the root should occur. I think that under-lying it all is the question of the status of the patient's nutrition, whether it is in a condition of equilibrium, or whether the balance is upon the side of repair or that of atrophy. I think the nutritive conditions are variable in the individual from time to time, and that may account for the favorable or unfavorable results obtained in the operation. It is exceedingly difficult to determine what the condition of these nutritive processes is at any given time. We have a familiar example in cases of pyorrhœa alveolaris, where the patients are otherwise in a normal condition, and yet there is this local expression of what I regard as a systemic vice, which seems to depend upon some nervous nutritive control of the mucous membrane and ligamentous tissues of the mouth that has its expression only there. We find an atrophic variety of nasal catarrh, that being only a local expression of some error in the system, and which seems to be of nervous origin. The trophic centres presiding over those tissues seem to be in an abnormal condition. Just why one cell builds up and another breaks down, and just what the conditions are which control the process, it is difficult, if not impossible, for us to decide in our present state of knowledge; and until we do know more about it, and can select our subjects with more care and intelligence, we will have these cases of absorption of the root, no matter how careful we may be in the operation.

In general, it seems to me that there is a future for this operation that is full of promise, notwithstanding the percentage of failures that have been reported.

*Dr. De Lange*—I had the opportunity of witnessing some of Dr. Younger's operations in San Francisco, in December, 1886. With regard to the peridental membrane, Dr. Younger says he performed the operation of implantation in several cases where the tooth was denuded of its peridental membrane, and those cases were invariably failures.

*Dr. Ivory*—In two cases operated upon I have had success. A lady came into my office one day having a lower cuspid tooth on the right side with quite a large cavity in it, and she wanted it removed. She was a poor woman, and could not afford to have any filling done. I extracted the tooth and told her to wait a few minutes. I took the tooth into my laboratory and cut off the root, and drilled it out and filled it partly up with cement; then filled the root with gold to the apex, flushing it all over and around the end of the root. I filled the other part with cement. I replaced the tooth in the mouth merely as an experiment, telling her to come back in two weeks. She did so, and it seemed to be as firm as when it was put in. I cannot say how it is now, or whether there is anything more than a mechanical union.

*Dr. Sudduth*—I have had no practical experience myself in this operation; but I have studied somewhat the histological and pathological points that bear upon it, and I look very favorably upon the results to be obtained. The subject of implantation is not a new one, although it has been brought recently into prominence. I think that with our present knowledge of how to control inflammatory processes, and especially septic processes, we will have much better results from the operation than could have been obtained or expected in the earlier experiments in this direction. As regards the method of attachment, there is no doubt at all but what it is at first one of encapsulation, and following that, if the inflammatory conditions are kept in abeyance, we may get a bony anchylosis. The pericemental attachment of the teeth serve as a cushion between the root of the tooth and the bone, which in masticating serves to soften the blow. In the case of an implanted tooth we have no such fibrous tissue between, and if it is a case of bony anchylosis, the bone and the tooth come directly in contact, there is a different condition from that of a natural tooth, a condition which can be detected by the sound. If you tap with an instrument an implanted tooth it gives a hard resonant sound that is not perceived when a natural tooth in its original condition is tapped in the same way. There evidently is no intervening membrane between the tooth root and the bone; and we must infer that such a tooth would be a weaker member than one which has been undisturbed. I think it is decidedly weaker as a bridge work post than a normal root would be, and great care should be taken not to place too much strain upon such teeth.

The operation is comparatively painless. If proper antiseptic precautions are taken there is very little or no danger of impregnating the surrounding tissue with disease.

The statement is made that a tooth had been implanted with success which had upon the end of its root calcific deposits. Implanting a tooth in that condition would, it seems to me, be almost criminal. I think that all antiseptic precautions should be taken before this operation is performed.

Another question in this connection that has been talked of between Dr. Kirk and myself is how far is a dentist justifiable in proceeding in surgical operations of this kind, in which disease may possibly follow, and how far is he protected by law in these operations. It would surely place adentist in a bad light if he were to be called before a grand jury to give expert testimony in regard to this matter, and he should be found to know nothing about antiseptic conditions and the means of preventing them. If the testimony showed that the dentist had implanted a tooth that had not been thoroughly antiseptized it would place him in a very bad position indeed, although he might be perfectly competent to perform the mechanical operation. But are we competent to take charge of such a case from its medical aspect?

*Dr. Dwinelle*—I cannot speak from a very large experience on this subject; mine has been very limited; but I was intimately associated with Dr. Younger in the early part of his career here, and took great interest in his operations. I had great incredulity that such operations could be successfully performed, because it is really contrary to our prior knowledge of anatomy and physiology and the healing art; nevertheless, if a person can prove that he has jumped over the moon we are bound to believe it.

I want to sound a word of caution for the benefit of our ship of state in regard to the anæsthetic part of this subject, with particular reference to cocaine. The use of this drug up to this date is entirely experimental. We know that there are individual idioysncrasies of constitution, and that an article which one person may take with impunity would be exceedingly deleterious, if not fatal, to another. You know that some people who are in the habit of taking morphine or opium can take enough of this drug to kill half a dozen other men. These idiocyncrasies are peculiar to the individual. I have had some experience with cocaine, and also with its parent, cocoa leaves, and I take great interest in it. I have seen several instances of the administration of cocaine where there was some doubt about the recovery of the patient from its effects. So I merely rise to sound a word of alarm, and to issue a caveat to our profession at large to be more cautious in the administration of this drug. I think the best possible safeguard in the use of cocaine is to fix a limit to the quantity administered, and not to exceed that limit. I was especially delighted with the operation of Dr. Kirk here, when I found that he put a limit upon the quantity of cocaine, $\frac{1}{8}$ grain, used. We should all do that, especially as we know so little about this agent. He passed the point of the hypodermic syringe up between the periosteum and the alveolar process externally, on the labial surface, about half an inch, introduced his eighth of a grain and let it remain for five minutes or a little longer before he commenced the operation, which was then comparitively painless. I have seen dentists use cocaine *ad libitum* in operating; they were continually injecting it from the beginning to the end of the operation. It seems to me that discretion would be the better part of valor, for I have seen instances where a patient has manifested very serious and alarming symptoms.

*Dr. Kirk*—Mr. President, I will speak a word on the subject of cocaine. I have always pursued the plan of using a definite quantity of cocaine, because frequently we get too profound an effect from it. I have been led to the use of one-eighth grain doses as a fair average; and from my study of the subject, I think its physiological action has been pretty well settled. I think perhaps one of the best investigations that has been made upon the subject was made by Dr. Hugenschmidt, of Paris, an associate of Dr. Thomas Evans. I have had the opportunity of looking over his work. He has made a quite extended investigation of its physiological action, and has used it largely for the purpose of extracting and in the minor surgery of the mouth, and he has said distinctly that its action is that of a vaso-motor constrictor. Knowing this to be its action, the antidote that suggests itself is nitrate of amyl, which acts in an entirely opposite direction. I have had one or two instances of somewhat marked systemic disturbance from the use of cocaine, and have counteracted it by the use of nitrate of amyl. It is one of the most potent remedies against syncope or heart failure, and controls the influence of cocaine at once. The effect of the nitrate of amyl upon the heart is to stimulate its action. Hypodermic injections of ether, or the exhibition of ether by the stomach; hot brandy punches, or hypodermic injections of ammonia, or the exhibition of aromatic spirits of ammonia by the stomach, may be resorted to as means for controlling the effect of an overdose of cocaine. A heart stimulant is what is needed. While some fatal cases have been reported, it is to be noted that as much as one grain of cocaine has been given hypodermically without fatal results. We should know the limitations of a drug in order to use it intelligently.

*Dr. Cornwall*—With regard to what has been said about waiting five or six minutes for the cocaine to have its effect; I witnessed a clinic given by Dr. Perine, in which he commenced to operate immediately after the administration of the cocaine, he claiming that the effect was instantaneous.

*Dr. Dwinelle*—It is not instantaneous.

*Dr. Cornwall*—There has been some curiosity expressed by those who witnessed the operation in my case as to the amount of pain endured. I believe most of those who saw the operation know that there was no cocaine administered; but this morning my attention was called to an article in one of the local papers, which stated that I was enabled to bear the pain through the application of Christian Science However that may be, I am not prepared to state positively that that helped me or that it did not. I have never had any experience before this with the Mind Cure or Christian Science. But I am not prejudiced against it, and I don't know anything in relation to it to make me very highly in favor of it. In fact I do not know of any way to settle that point, as to whether it was of any benefit to me or not in the operation other than having some teeth put in without the aid of Christian Science and without cocaine. I have a space left that is not yet filled, and if Dr. Kirk thinks it justifiable I am willing to try it. I am ready to give this a fair test, and I do it quite as much for my own satisaction as for any other purpose.

*Dr. Sudduth*—Is there any soreness to-day around the implanted tooth?

*Dr. Cornwall*—There is no soreness. It is very quiet.

*Dr. Wheeler*—What was the difference between this process and the cutting of sensitive dentine?

*Dr. Cornwall*—I think cutting the sensitive dentine is more sharply felt than cutting a socket in the bone.

*Dr. Kirk*—The experience of Dr. Cornwall as to the amount of pain he suffered differs not at all from that of the patients for whom I have operated without any anæsthetic. It is not a painful operation by any means. I am sorry that this question of Christian Science has been brought in, in this connection. I am pleased that Dr. Cornwall has expressed his views upon the subject and I will state for myself that there is joy amongst one dentist in this convention over one sinner that has repented.

*Dr. Smith*—There is one thing in which I differ from some others: that is in being careful to have the tooth fastened in thoroughly so that there is no chance of displacing it. The tooth which I first saw implanted by Dr. Younger, in Rochester, was a bicuspid, and the fellow went to a political meeting three or four days afterwards and got it knocked out. It was tied in with a little thread. The manner in which I fasten these teeth in is this: if there is no cavity in the tooth I cut one, and also cut one in the adjoining tooth, and put in a small staple running from one to the other, cementing it in, which holds the implanted tooth securely. It is left for two or three months until the tooth becomes firm.

*Dr. Curtis*—I think, like Dr. Kirk, that it is unfortunate that Christian Science has been mixed up with us here, and to have it go out to the world that we tolerated and gave some sort of endorsment, to Christian Science. I believe Christian Science is an imposition, and I do not think there is a man in this room who believes that it is anything more than an old woman's whim.

I read my paper here under protest. I got it up in a few hours, doing the best I could in that time. I am glad to have seen the operations yesterday, and I hope there are many more converts to implantation. As Dr. Sudduth says, unless we study the subject, and select our cases with great care, we will have failures and may have to suffer if brought before the law if we are not thoroughly posted in the matter.

As to cocaine, I believe we can very readily get a toxic effect with it. I have in a number of cases seen it in my patients. One was the case of my brother, a physician, who studied the physiological action of the cocaine. In two and a half minutes he lost his suffering. It made him sick at the stomach. He said that a minute after the injection he was stimulated to the extreme, and in three or four minutes after he was relieved. I injected it directly into a bloodvessel. I have not seen any really bad effects from the use of it. I never give it except where the patient demands it. I think a person is better qualified to stand pain without the drug than with it, and in most cases I do not use it at all.

The paper was passed.

